# Road-traffic noise exposure and coronary atherosclerosis in the Swedish CArdioPulmonary bioImage Study (SCAPIS)

**DOI:** 10.1097/EE9.0000000000000344

**Published:** 2024-10-03

**Authors:** Marat Murzabekov, Åsa Persson, Christian Asker, Karl Kilbo Edlund, Charlotta Eriksson, Tomas Jernberg, Peter Molnar, Anna Oudin, Andrei Pyko, Jenny Lindvall, Mare Lõhmus, Kerstin Persson Waye, Johan Nilsson Sommar, Leo Stockfelt, Mårten Spanne, Magnus Svartengren, Mikael Ögren, Göran Pershagen, Petter Ljungman

**Affiliations:** a Institute of Environmental Medicine, Karolinska Institutet, Stockholm, Sweden; b Swedish Meteorological & Hydrological Institute, Norrköping, Sweden; c Occupational and Environmental Medicine, School of Public Health and Community Medicine, Institute of Medicine, Sahlgrenska Academy, University of Gothenburg, Sweden; d Department of Occupational and Environmental Medicine, Sahlgrenska University Hospital, Gothenburg, Sweden; e Centre for Occupational and Environmental Medicine, Region Stockholm, Stockholm, Sweden; f Department of Clinical Sciences, Danderyd University Hospital, Karolinska Institutet, Stockholm, Sweden; g Department of Public Health and Clinical Medicine, Faculty of Medicine, Umeå University, Umeå, Sweden; h Division of Occupational and Environmental Medicine, Department of Laboratory Medicine, Faculty of Medicine, Lund University, Sweden; i SLB-analys, Environment and Health Administration, Stockholm, Sweden; j Environment Department, City of Malmö, Malmö, Sweden; k Department of Medical Sciences, Occupational and Environmental Medicine, Uppsala University, Uppsala, Sweden; l Department of Occupational and Environmental Medicine, Uppsala University Hospital, Uppsala, Sweden; and; m Department of Cardiology, Danderyd Hospital, Stockholm, Sweden

**Keywords:** noise, atherosclerosis, road traffic noise, cardiovascular disease

## Abstract

**Background:**

Road-traffic noise may influence the development of cardiovascular events such as stroke and myocardial infarction, but etiological mechanisms remain unclear. This study aimed to assess the relationship between long-term road-traffic noise exposure and coronary atherosclerosis in Sweden.

**Methods:**

In the Swedish CArdioPulmonary bioImage Study (SCAPIS) cohort, including 30,154 subjects aged 50–65 years, recruited between 2013 and 2018, coronary atherosclerosis was measured based on computer tomography (CT) scans as coronary artery calcium score, segment involvement score (SIS), and non-calcified plaques (NCP) at enrollment. Based on modified Nordic model, road-traffic noise exposure was modeled for 2000, 2013, and 2018 with interpolation for intermediate years. We investigated the association between time-weighted long-term exposure to road-traffic noise (L_den_) and the prevalence of atherosclerosis using ordinal logistic regression models adjusting for potential socioeconomic, behavioral, and environmental confounders, including air pollution.

**Results:**

No clear associations were found between road-traffic noise and coronary atherosclerosis. The odds ratio for coronary artery calcium score was 1.00 (95% confidence interval [CI] = 0.96, 1.04), SIS 0.99 (0.96, 1.03), and NCP 0.98 (0.90, 1.03) per interquartile range (9.4 dB L_den_) for road-traffic noise exposure during 10 years before enrollment. No consistent associations were observed in site-specific analyses or using shorter exposure periods. Furthermore, exposure-response analyses revealed no clear trends, and there were no strong interactions between road-traffic noise and cardiovascular risk factors in relation to the atherosclerosis markers.

**Conclusions:**

Long-term exposure to road-traffic noise was not linked to coronary atherosclerosis or calcification in relatively healthy, middle-aged populations in Sweden.

**What this study adds**

While noise exposure has been associated with the risk of having a myocardial infarction or stroke, mechanisms remain unclear. Our cross-sectional study investigated the relationship between noise exposure and coronary atherosclerosis in a large general population sample using high-quality imaging techniques. We did not find clear or consistent associations between exposure to road-traffic noise and three different measures of coronary atherosclerosis. These findings contribute to the understanding of possible mechanisms behind noise-related ischemic heart disease and stroke.

## Introduction

Continuing urbanization leads to increases in the number of people exposed to road-traffic noise, and a substantial proportion of the urban population in Europe is exposed to noise levels considered harmful to health.^1,2^ There is growing evidence that road-traffic noise is associated with increased risk of cardiovascular disease, including ischemic heart disease and stroke.^2,3^ However, there is limited understanding of specific pathophysiological mechanisms for how noise exposure can contribute to the development of cardiovascular disease. Evidence shows that noise-induced stress and sleep disturbance lead to the activation of autonomic and endocrine systems, which, in turn, contribute to elevated blood pressure, oxidative stress, and endothelial dysfunction.^3,4^ These physiological reactions could contribute to the development of atherosclerosis, including atherosclerosis in coronary arteries, which is a precondition for ischemic heart disease and acute coronary events such as myocardial infarction.^5^ However, only a few studies have investigated the association between road-traffic noise and atherosclerosis,^6–8^ with inconsistent results, and more importantly, no prior study has specifically investigated coronary atherosclerosis.

The assessment of atherosclerosis in coronary arteries through coronary computed tomography angiography is a method with very high sensitivity for the detection of coronary artery disease.^9^ Coronary computed tomography angiography can be used to measure coronary atherosclerosis in several ways. The widely used Agatston’s coronary artery calcium score (CACS) takes into consideration atherosclerotic plaque area and calcium lesion density. High CACS is a predictor of future cardiovascular events in asymptomatic individuals.^10^ While CACS considers only calcified plaques, segment involvement score (SIS) quantifies the number of coronary segments with atherosclerotic plaques, assessing the extent of the disease without focusing on the composition of the plaques.^11^ A third method quantifies the presence of non-calcified plaques (NCP), suggested as a marker of vulnerable plaques prone to lesions and subsequent cardiovascular events.^12^ These markers of coronary atherosclerosis have only been studied to a limited extent in relation to environmental exposures such as air pollution, noise, and access to green spaces.^13^

In this study, we investigated the association between long-term road-traffic noise exposure and coronary atherosclerosis in a large population-based cohort of middle-aged men and women. Our main hypothesis was that higher exposure to noise would be associated with atherosclerosis marker scores, such as CACS, SIS, and NCP.

## Methods

### Study participants

The study participants were selected from the Swedish CArdioPulmonary bioImage Study (SCAPIS), which is an ongoing population-based cohort study targeting middle-aged individuals. The study included 30,154 men and women (aged 50–65 years) randomly selected from six Swedish cities (Gothenburg, Linköping, Malmö, Stockholm, Umeå, and Uppsala). The recruitment took place between 2013 and 2018. The collected data included detailed computer tomographic imaging (including computed tomographic angiography), blood samples, and responses to questionnaires regarding the participants’ background characteristics and behaviors. Further details on cohort design, participants, recruitment, and data collection have been described elsewhere.^14,15^

We obtained coordinates of all residential addresses during the 10 years before recruitment for all SCAPIS participants and linked these to environmental data for estimation of individual exposure. Ethical approval was obtained for the SCAPIS study (DNR 2010-228-31M) and for this study (DNR 2019-06469).

### Road-traffic noise

Road-traffic noise levels were modeled for 2000, 2011, and 2018 with interpolation for intermediate years.^16^ Input data included 3D terrain data, information on the ground surface, road network, daily traffic flows, speed limits, and percentage of heavy vehicles. To calculate noise levels for road traffic a modification of the Nordic prediction method was used, where possible reflection and shielding were taken into account by a Ground Space Index based on building density. The methodology has been further developed from the one described by Ögren and Barregard,^17^ which was validated against the full Nordic prediction method modeled with SoundPlan and showed coherent estimates. Noise maps were created for the study areas with a spatial resolution of 25 × 25 m in the most densely populated areas, while in sparsely populated areas the resolution was 100 × 100 m.

For this study, the calculations of L_Aeq,24h_ were converted to the European Union noise indicator day-evening-night (L_den_), which penalizes noise occurring during evening and nighttime. L_den_ was calculated by adding 3 dB to L_Aeq,24h_, based on typical Swedish road traffic time distributions.^17^ Participants missing exposure data for more than 2 of 10 years before enrollment were excluded from the analysis. Noise values below 40 dB were set to 40 dB L_den_, due to considerable imprecision in estimates for values below this level.^18^ Long-term exposure to noise was modeled as time-weighted (energy-weighted) means considering changes in residential addresses. We modeled the average exposure for 10, 5, and 2 years preceding enrollment, where 10 years average was our a priori exposure window of interest since atherosclerosis is a slowly progressive disease.

### Air pollution

Air pollution was modeled for each study site using methods described elsewhere.^19^ In brief, emission inventories for all study sites were compiled for PM_2.5_, PM_10,_ and NOx for the years 2000, 2011, and 2018. Concentrations of source-specific pollutants were modeled using high-resolution dispersion models with grids of 50 × 50 m for the most densely populated areas and up to 500 × 500 m for the least populated areas. Concentrations of long-range transported pollutants were estimated with bias-corrected chemical transport modeling on a 15 km grid. The air pollution values for the intermediate years were interpolated and adjusted based on meteorological data. Based on individual time-variant address data, each study participant was assigned the average residential exposure for each year between 2000 and 2018, with subsequent estimation of time-weighted exposures during the relevant time period. We observed high correlations between NO_X_ and NO_2_ levels (*r* = 0.99) and used NO_2_ in the adjustment models to facilitate comparisons with other studies.

### Greenness

Exposure to residential greenness was estimated using the annual maximum values of the normalized difference vegetation index (NDVI) derived from satellite data with a spatial resolution of 25 × 25m.^13^ To reduce the effect of cloud contamination a 5-year floating average value was used, based on the pixel values for the actual year, 2 years before, and 2 years after a given year. The research participants were attributed a median NDVI value for all nonwater surfaces within circular buffers with 500 m radii centered at the coordinates of the residential addresses.

### Measurement of calcification and atherosclerosis

Upon recruitment, the participants underwent cardiac computed tomography (CT). The image acquisition methodology and subsequent assessment of atherosclerosis and calcification in SCAPIS have been described previously.^15^ In brief, the assessment of coronary calcium included using non-contrast-enhanced, retrospectively electrocardiogram-gate multisection CT scanner (Somatom Definition Flash, Siemens Medical Solutions, Gothenburg, Sweden).

Calcification was assessed in four coronary arteries: the circumflex coronary, the left main, the left anterior descending, and the right coronary arteries. Calcified areas were summed up for all arteries and slides and then multiplied by an intensity factor to produce a total CACS.^20^ In line with previous research, we classified CACS as none (0), ultralow (1–10), low (11–100), moderate (101–400), or high (>400).

Additional measures of the extent of coronary atherosclerosis disease (SIS and NCP) were obtained through visual scoring by a group of trained thoracic radiologists and cardiologists. Coronary segments were visually assessed to identify plaques and to classify plaques as calcified or noncalcified, as well as to define the level of stenosis. It was compulsory to report on 11 clinically most relevant segments. The remaining segments were reported only if they had atherosclerosis.^15^ The number of segments with any stenosis represented the SIS.^21^ NCP, on the other hand, was defined as the number of coronary segments with stenosis but without detectable calcification.

### Covariates

Based on self-reported data about smoking habits, the study participants were categorized as: “never-smokers,” “ex-smokers,” and “current smokers.” Leisure-time physical activity was categorized as mainly sedentary activity such as reading or watching television, moderate activity such as walking or cycling for at least 4 hours/week, regular strenuous physical activity, and athletic training several times per week. Alcohol consumption was categorized as the self-reported frequency of consuming an alcoholic drink during the last year. Civil status was divided into four categories: “living alone,” “married,” “divorced,” and “widow.” Hormone-replacement therapy was a binary variable indicating ever-use for menopausal symptoms. Foreign/Swedish background was categorized into: “Swedish born” or “foreign born.” Education was divided into three categories: “primary school,” “secondary school,” and “university education or higher.” Based on data obtained from Statistics Sweden for 2000, 2011, and 2018, area-based socioeconomic status was assigned to each individual using the percentage of individuals with an income below the lowest Swedish income quartile for each Demographic Statistics Area unit.

### Statistical analyses

Associations between long-term exposure to road-traffic noise and the three outcome variables (CACS, SIS, and NCP) were estimated using ordinal logistic regression. Different sets of variables were used in a stepwise manner, including known cardiovascular risk factors, which may also be related to road-traffic noise exposure and thus constitute confounders. Exposure to noise was modeled as a time-weighted mean over 10-year periods preceding the enrollment. All risk estimates were presented per interquartile range (IQR) increase (9.4 dB L_den_). The first regression model included age, gender, and enrollment site. We added the following variables for the second model: neighborhood proportion of low-income earners, highest attained education level, cohabitation or civil status, use of hormone-replacement therapy for menopausal symptoms, and country of birth. The third model (main model) additionally included behavioral risk factors: smoking status, leisure-time physical activity, and alcohol consumption. We analyzed only the participants with complete data for the main model to ensure comparability across models. For exposure-response analysis, we replaced the continuous exposure variable with a restricted cubic spline with three degrees of freedom (knots at 5th, 35th, 65th, and 95th percentile).

We analyzed effect modification by introducing an interaction term between the potential effect modifier and the 10-year noise exposure. The analysis focused on sex, age (using five-year categories), smoking status, use of lipid-lowering statins, body mass index (BMI), serum level of low-density lipoprotein cholesterol (calculated using Friedewald’s formula and dichotomized at the cohort median), high-sensitivity C-reactive protein (dichotomized at a serum level of 3 mg/L). Wald test was used to calculate the *P* values of interaction.

In sensitivity analyses, we added exposure to air pollution, defined as annual concentrations of PM_2.5_, PM_10_, and NO_2_ averaged over 10 years, to the main model. We also tested the impact of adding greenness (NDVI) averaged over 10 years to our main model. Furthermore, we ran models using road-traffic noise exposure averaged over 2- and 5-years before enrollment. We also tested the impact of adding urbanicity (based on the classification of demographic statistical areas used by Statistics Sweden), employment status, and BMI. BMI may be a confounder of the association between road-traffic noise exposure and coronary atherosclerosis but may also be a mediator in a causal pathway between noise exposure and atherosclerosis. If BMI acts as a mediator, it should not be included as an adjustment variable.

All analyses were performed in R 4.2.1 using the *rms* package.^22^

## Results

From the original sample of 30,154 individuals, 1111 (3.7%) were excluded due to missing exposure data and 2378 (7.9%) due to missing covariate data. Data from 26,665 participants, with complete covariate information, was included in the main model (Figure S1; http://links.lww.com/EE/A307). Overall, 51.5% were women, mean age was 57 years, 12.1% were smokers, 42.3% had detectable coronary artery calcifications, and 16% had detectable noncalcified plaques.

There was a higher prevalence of atherosclerosis among men than among women (56% of men vs. 29% of women had SIS ≥1). Atherosclerosis was more prevalent in older participants than in younger ones (29% among 50–54 years old had SIS ≥ 1, while 53% among 60–65 years old). Exposure to road-traffic noise varied between the sites (Figure S2; http://links.lww.com/EE/A307). Mean 10-year exposure to noise was highest in Malmö (59.5 dB L_den_) and lowest in Umeå (52.1 dB L_den_). The mean values for other cities were: Gothenburg (58.9 dB L_den_), Linköping (53.9 dB L_den_), Stockholm (57.8 dB L_den_), and Uppsala (52.3 dB L_den_). The WHO guideline value of 53 dB L_den_ was exceeded for 71.7% of the participants. Mean exposure during 10 years before enrollment was highly correlated with mean 5-year and 2-year exposure before enrollment (all *r* ≥ 0.9). There was a moderate positive relationship between road-traffic noise and PM_2.5_ exposure (*r* = 0.43), and a moderate negative relationship between noise and greenness exposure (*r* = −0.6) (see Table S1; http://links.lww.com/EE/A307).

As shown in Table 1, participants with higher exposure to noise were more likely to smoke, live alone or be unmarried, consume alcohol more frequently, be foreign-born, have lower educational level, and reside in areas with a higher percentage of low-income earners. The study sites differed in terms of the prevalence of current smoking (from 8% in Umeå to 16.5% in Malmö), frequent drinking (from 3% in Umeå to 10% in Stockholm), and the proportion of low-income earners (from 20% in Stockholm to 30% in Malmö). Participants excluded because of missing exposure or covariate data were more likely to be foreign-born (27.6%), have lower education level (15.3%), more likely to be active smokers (25%), and have a higher prevalence of atherosclerosis (48% SIS ≥1), calcification (46% CACS ≥1), and NCP (14% NCP ≥1).

**Table 1.** Participant characteristics, grouped by quarter (Q1–4) of mean total L_den_ (dB) exposure to road-traffic noise 10 years before enrollment

	Q1	Q2	Q3	Q4
L_den_, mean	46	54.81	59.44	65.25
N	6667	6666	6666	6666
Demographical indicators				
Age, mean	57.4	57.5	57.4	57.6
Gender, % female	49.3	51.9	52.5	52.5
Low-income proportion, mean area-level % of low-income earners	19.7	23.3	25.8	26.9
Education, % with only compulsory schooling or less	8.2	8.6	9.3	9
Civil status, % living alone or unmarried	6.7	9.6	16	21.2
Hormone-replacement therapy, % of women ever-users for menopausal symptoms	21.9	22.5	25.9	25.9
Foreign-born, % not born in Sweden	9	13.5	18.4	19
Behavioral cardiovascular risk factors				
Smoking, % current smokers	8.3	10.7	13.3	16.1
Low physical activity, % reporting no regular leisure-time exercise	10.3	11	12.9	13
Alcohol consumption, % consuming alcohol more than once a week	37.1	37.3	37.3	40.3
Co-exposures (averaged over 10 years before enrollment)				
Air pollution, mean PM2.5	5.6	6.4	6.9	7.4
Greenness, mean NDVI within 500 m	0.6	0.5	0.5	0.4
Cardiovascular outcomes				
Calcification, % CACS ≥1	39.8	39	40.9	41.2
Non-calcified plaques, % any plaques	11.6	11.1	10.9	11.4
Stenoses, % SIS ≥1	41	41	43	44

**Figure 1.**
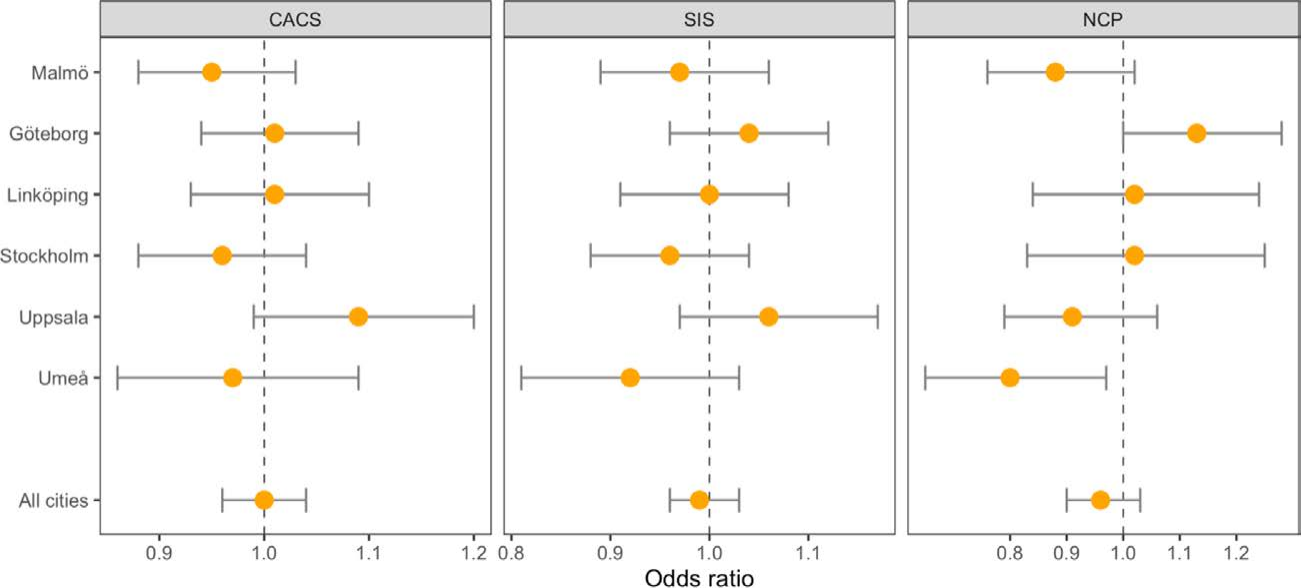
Site-specific associations between mean road-traffic noise exposure for the 10 years preceding the enrollment and atherosclerosis, including CACS, SIS, and NCP.

Road-traffic noise averaged over 10 years at the residential address was not associated with any of the measures of atherosclerosis in our main analysis (Table 2). For the main model, the odds ratio for CACS was 1.00 (95% CI = 0.96, 1.04), SIS 0.99 (95% CI = 0.96, 1.03), and NCP 0.96 (95% CI = 0.90, 1.03). We observed no clear heterogeneity between recruitment sites, except for NCP, where noise exposure was positively associated with NCP in Gothenburg (OR = 1.13; 95% CI= 1.00, 1.28) and negatively associated in Umeå (OR = 0.80; 95% CI = 0.65, 0.97 per IQR increase in L_den_) (Figure 1).

**Table 2.** Odds ratio and 95% CI for atherosclerosis in relation to exposure to road-traffic noise per IQR (9.42 dB L_den_) during 10 years before enrollment

	Coronary artery calcium score (CACS)	Segment involvement score (SIS)	Non-calcified plaques (NCP)
Model 1^a^	1.03 (1.00, 1.07)	1.03 (0.99, 1.07)	0.97 (0.91, 1.04)
Model 2^b^	1.02 (0.98, 1.06)	1.01 (0.97, 1.05)	0.97 (0.90, 1.03)
Model 3 (main)^c^	1.00 (0.96, 1.04)	0.99 (0.96, 1.03)	0.96 (0.90, 1.03)

a Model 1 included age, gender, and recruitment site.

b Model 2 also included area-level socioeconomic status, education level, cohabitation or civil status, hormone-replacement therapy, and country of birth.

c Model 3 also included smoking status, physical activity, and alcohol consumption.

**Figure 2.**
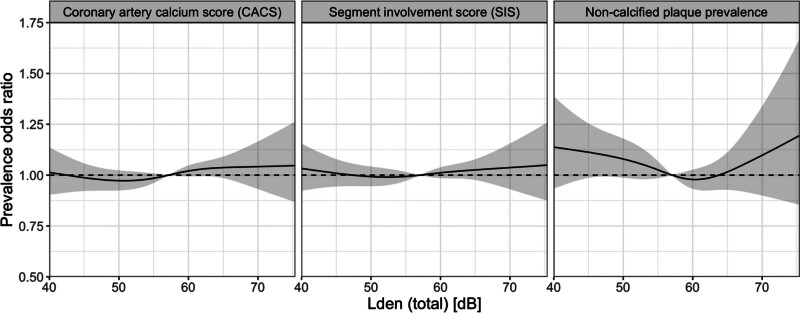
Restricted cubic splines with three degrees of freedom for exposure to road-traffic noise (dBL_den_), in the main covariate model, with 95% confidence bands.

The exposure-response analysis showed no consistent patterns for CACS, SIS, or NCP (Figure 2). A U-shaped function was suggested for NCP, but there was substantial uncertainty at high and low exposure levels because of low numbers.

**Figure 3.**
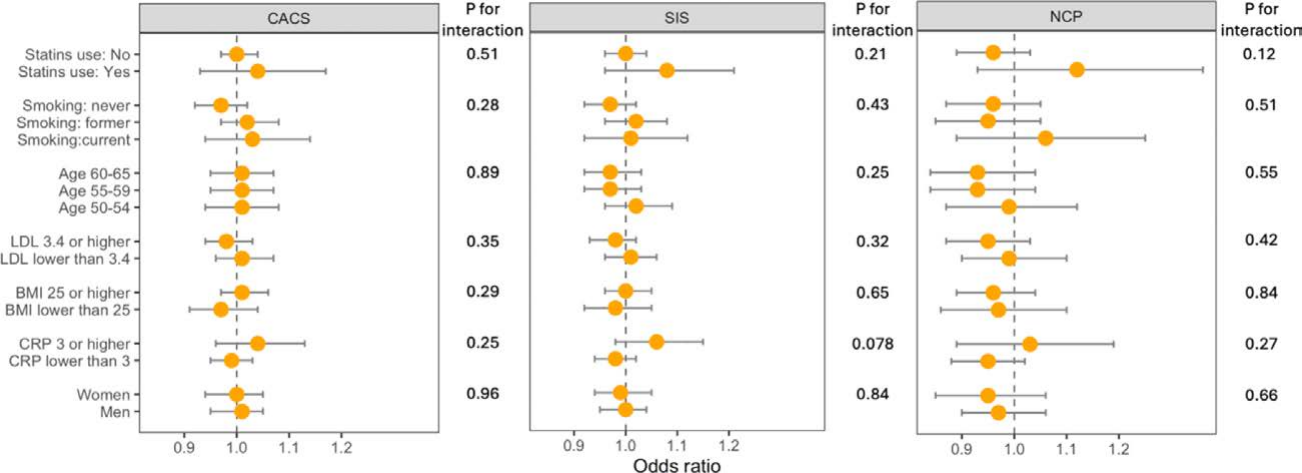
Odds ratio and 95% CI for atherosclerosis in relation to exposure to road-traffic noise per IQR increase during 10 years before the enrollment according to strata of potential effect modifiers. Analyses based on separate models with interaction terms between road-traffic noise and each potential modifier, adjusted for age, sex (men/women), educational level (low/medium/high), marital status (single/married), area-level socioeconomic status, smoking status, physical activity, and alcohol consumption.

Effect modification analysis of the association between road-traffic noise and three outcome measures of atherosclerosis did not demonstrate consistent evidence of differing associations across covariates (Figure 3).

Sensitivity analyses focusing on atherosclerosis are presented in Figure S3; http://links.lww.com/EE/A307. The adjustment for covariates had limited effects on ORs and gave nearly identical results for all outcomes. Adjustment for PM_2.5_ or NO_2_ resulted in minor changes to risk estimates compared with the main analyses except that adjustment for PM_2.5_ in analyses of the association between road-traffic noise and noncalcified plaques showed a reduced risk of noncalcified plaques (OR = 0.91; 95% CI = 0.85, 0.98). Adjusting for employment status and urbanicity resulted in minor changes to risk estimates. Finally, the adjustment for BMI had limited effects for the CACS and SIS models, while marginally decreasing the point estimate for NCP. Modeling road noise exposure using alternative averaging periods resulted in consistently null results regardless of the averaging period.

## Discussion

We investigated associations between long-term road-traffic noise exposure at the residential address of 26,665 middle-aged participants from six Swedish cities and three measures of coronary atherosclerosis. We did not observe consistent evidence of associations between road-traffic noise and any of the measures of atherosclerosis. Results were robust to multiple sensitivity analyses.

No study has previously specifically considered coronary atherosclerosis, however, some previous research has investigated the association between road-traffic noise and other measures of atherosclerosis with inconsistent results. A cross-sectional analysis of the German Heinz Nixdorf Recall cohort study found an association between L_night_ and atherosclerosis, where a 5 dB increase was associated with a 3.9% increase in thoracic aortic calcification (95% CI = 0.0, 8.0).^8^ However, a more recent longitudinal update of the German Heinz Nixdorf Recall study demonstrated no overall association but found an increased risk of developing subclinical atherosclerosis related to noise exposure in participants who had no or minimal thoracic calcification at baseline.^7^ Using carotid intima-media thickness as a proxy for atherosclerosis, the WhiteHall II and Southall and Brent REvisited cohort demonstrated a positive association in between nighttime noise exposure and carotid intima-media thickness.^6^

Other studies have reported associations between road-traffic noise exposure and possible consequences of coronary atherosclerosis, such as myocardial infarction. Two meta-analyses of road-traffic noise and myocardial infarction reported positive associations, but confidence limits often spanned the null except in cross-sectional or case–control studies, limiting the level of evidence.^23,24^ However, recently published studies in large cohorts such as the national cohort of Denmark,^25,26^ the Danish Nurses Cohort^27^ or multicenter cohorts in Denmark, Sweden and Finland^18^ have reported higher risk of incident myocardial infarction or a broader category of ischemic heart disease with long-term road-traffic exposure. In a large population-based cohort in 1 million inhabitants of Toronto, Canada road-traffic noise was also associated with incident myocardial infarction.^28^ In the Swiss national cohort, road-traffic noise was associated with a higher risk of mortality due to myocardial infarction.^29^ As a whole, there is growing evidence to support the role of road-traffic noise exposure in increasing the risk of myocardial infarction yet very limited attempts to investigate coronary atherosclerosis, a cornerstone of ischemic heart disease, as a pathway of effect. In the absence of evidence of associations between noise exposure and atherosclerosis, other etiological pathways might be explored, such as involving the risk of plaque rupture independently of plaque formation through noise-induced stress mechanisms.^4^

One possible explanation for our null findings is imprecision in the exposure assessment. We assigned modeled road-traffic noise exposure levels at the façade but did not have information on the time spent indoors or indoor noise levels.^30^ Indoor noise levels can vary depending on apartment orientation and the quality of sound insulation, which depends on the buildings’ age, and targeted noise insulation measures. We also lacked information on other noise sources, that is, railway and aircraft noise. This could have led to misclassification of exposure and bias towards the null in analyses of associations between exposure and effects.

The risk of atherosclerosis did not change significantly after adjustment for potential confounders, including air pollution and greenness. This is consistent with the earlier research that air pollution and the lack of green space do not seem to confound the associations between road-traffic noise and cardiovascular health.^25,31^ The risk estimates for NCP decreased after the adjustment for air pollution, but the percentage change in estimate was less than 10%.

There are several limitations to this study. The main limitation is the cross-sectional nature, which limits opportunities to draw conclusions about causality since the temporal relation between the exposure and outcome is unclear. Another limitation is the partially subjective nature of the assessment of coronary atherosclerosis through SIS and NCP, which may have resulted in observation variability and biased measurements. Furthermore, noncalcified plaques were identified as stenotic coronary segments without any calcification. As a consequence, the methodology used did not identify partially calcified plaques (spotty calcification) or noncalcified plaques adjacent to calcified ones. This could have potentially led to a low prevalence of NCP in our study population. In addition, selection bias may have affected the results. There were indications that missing exposure or covariate data were related to low education, active smoking, and higher atherosclerosis markers. Since road-traffic noise exposure was also related to socioeconomic characteristics, nonresponse may have influenced the associations between noise exposure and coronary atherosclerosis. Furthermore, we did not have data on some of the factors affecting noise exposure, such as house characteristics (insulation and orientation of the rooms) or participants’ habits of keeping windows open at night. We also lacked information on noise annoyance and diet.

The strength of this study is the good quality data, including longitudinal address data, exposure data with high spatial and temporal resolution, extensive data on possible covariates, and well-characterized outcome data obtained through high-quality imaging techniques. The SCAPIS population is based on a random sample from six Swedish cities and is relatively representative of the middle-aged general population, although differences in acceptance rates based on socioeconomic status have been reported.^32,33^

## Conclusions

Overall, this cross-sectional study provided no clear or consistent evidence of an association between road-traffic noise and atherosclerosis. These results suggest that in a relatively healthy, middle-aged general population, road-traffic noise does not contribute to atherosclerosis or calcifications.

## Conflicts of interest statement

The authors declare that they have no conflicts of interest with regard to the content of this report.

## Supplementary Material

## References

[1] Exposure of Europe’s population to environmental noise. 2021 [cited 2023 Dec 30]. Available at: https://www.eea.europa.eu/en/analysis/indicators/exposure-of-europe-population-to-noise. Accessed 30 December 2023.

[2] Van Kempen E Casas M Pershagen G Foraster M. WHO Environmental Noise Guidelines for the European Region: a systematic review on environmental noise and cardiovascular and metabolic effects: a summary. Int J Environ Res Public Health. 2018;15:379. 29470452 10.3390/ijerph15020379 PMC5858448

[3] Münzel T Sørensen M Daiber A. Transportation noise pollution and cardiovascular disease. Nat Rev Cardiol. 2021;18:619–636. 33790462 10.1038/s41569-021-00532-5

[4] Münzel T Schmidt FP Steven S Herzog J Daiber A Sørensen M. Environmental noise and the cardiovascular system. J Am Coll Cardiol. 2018;71:688–697. 29420965 10.1016/j.jacc.2017.12.015

[5] Arbab-Zadeh A Nakano M Virmani R Fuster V. Acute coronary events. Circulation. 2012;125:1147–1156. 22392862 10.1161/CIRCULATIONAHA.111.047431 PMC3322378

[6] Halonen JI Dehbi HM Hansell AL. Associations of night-time road traffic noise with carotid intima-media thickness and blood pressure: the Whitehall II and SABRE study cohorts. Environ Int. 2017;98:54–61. 27712935 10.1016/j.envint.2016.09.023

[7] Hennig F Moebus S Reinsch N; Heinz Nixdorf Recall Study Investigative Group. Investigation of air pollution and noise on progression of thoracic aortic calcification: results of the Heinz Nixdorf Recall Study. Eur J Prev Cardiol. 2020;27:965–974. 31189380 10.1177/2047487319854818 PMC7272124

[8] Kalsch H Hennig F Moebus S; Heinz Nixdorf Recall Study Investigative Group. Are air pollution and traffic noise independently associated with atherosclerosis: the Heinz Nixdorf Recall Study. Eur Heart J. 2014;35:853–860. 24194529 10.1093/eurheartj/eht426

[9] Knuuti J Wijns W Saraste A; ESC Scientific Document Group. 2019 ESC guidelines for the diagnosis and management of chronic coronary syndromes. Eur Heart J. 2020;41:407–477. 31504439 10.1093/eurheartj/ehz425

[10] Gepner AD Young R Delaney JA. Comparison of coronary artery calcium presence, carotid plaque presence, and carotid intima-media thickness for cardiovascular disease prediction in the multi-ethnic study of atherosclerosis. Circ Cardiovasc Imaging. 2015;8:e002262. 25596139 10.1161/CIRCIMAGING.114.002262 PMC4299916

[11] Ayoub C Erthal F Abdelsalam MA. Prognostic value of segment involvement score compared to other measures of coronary atherosclerosis by computed tomography: a systematic review and meta-analysis. J Cardiovasc Comput Tomogr. 2017;11:258–267. 28483581 10.1016/j.jcct.2017.05.001

[12] Hou ZH Lu B Gao Y. Prognostic value of coronary CT angiography and calcium score for major adverse cardiac events in outpatients. JACC Cardiovasc Imaging. 2012;5:990–999. 23058065 10.1016/j.jcmg.2012.06.006

[13] Kilbo Edlund K Andersson EM Asker C. Long-term ambient air pollution and coronary atherosclerosis - results from the SCAPIS study. Atherosclerosis. 2024; 397:117576. 38797616 10.1016/j.atherosclerosis.2024.117576

[14] Bergström G Berglund G Blomberg A. The Swedish CArdioPulmonary BioImage Study: objectives and design. J Intern Med. 2015;278:645–659. 26096600 10.1111/joim.12384 PMC4744991

[15] Bergström G Persson M Adiels M. Prevalence of subclinical coronary artery atherosclerosis in the general population. Circulation. 2021;144:916–929. 34543072 10.1161/CIRCULATIONAHA.121.055340 PMC8448414

[16] Andersson EM Ögren M Molnár P Segersson D Rosengren A Stockfelt L. Road traffic noise, air pollution and cardiovascular events in a Swedish cohort. Environ Res. 2020;185:109446. 32278155 10.1016/j.envres.2020.109446

[17] Ögren M Barregard L. Road traffic noise exposure in Gothenburg 1975–2010. Bachschmid MM, editor. PLoS One. 2016;11:e0155328. 27171440 10.1371/journal.pone.0155328 PMC4865157

[18] Pyko A Roswall N Ögren M. Long-term exposure to transportation noise and ischemic heart disease: a pooled analysis of nine Scandinavian cohorts. Environ Health Perspect. 2023;131:17003. 36607286 10.1289/EHP10745 PMC9819217

[19] Kilbo Edlund K Kisiel MA Asker C. High-resolution dispersion modelling of PM2.5, PM10, NOx and NO2 exposure in metropolitan areas in Sweden 2000–2018 – large health gains due to decreased population exposure. Air Qual Atmos Hlth. 2024;17:1661–1675. Available at: https://link.springer.com/10.1007/s11869-024-01535-0.

[20] Agatston AS Janowitz WR Hildner FJ Zusmer NR Viamonte M Detrano R. Quantification of coronary artery calcium using ultrafast computed tomography. J Am Coll Cardiol. 1990;15:827–832. 2407762 10.1016/0735-1097(90)90282-t

[21] Min JK Shaw LJ Devereux RB. Prognostic value of multidetector coronary computed tomographic angiography for prediction of all-cause mortality. J Am Coll Cardiol. 2007;50:1161–1170. 17868808 10.1016/j.jacc.2007.03.067

[22] Harrell F Jr. rms: Regression Modeling Strategies. R package version 6.8-0. Available at: https://github.com/harrelfe/rms. Accessed 24 April 2024.

[23] Fu W Liu Y Yan S. The association of noise exposure with stroke incidence and mortality: a systematic review and dose-response meta-analysis of cohort studies. Environ Res. 2022;215:114249. 36058275 10.1016/j.envres.2022.114249

[24] Khosravipour M Khanlari P. The association between road traffic noise and myocardial infarction: a systematic review and meta-analysis. Sci Total Environ. 2020;731:139226. 32422434 10.1016/j.scitotenv.2020.139226

[25] Poulsen AH Sørensen M Hvidtfeldt UA. Concomitant exposure to air pollution, green space and noise, and risk of myocardial infarction: a cohort study from Denmark. Eur J Prev Cardiol. 2024;31:131–141. 37738461 10.1093/eurjpc/zwad306

[26] Thacher JD Poulsen AH Raaschou-Nielsen O. Exposure to transportation noise and risk for cardiovascular disease in a nationwide cohort study from Denmark. Environ Res. 2022;211:113106. 35304113 10.1016/j.envres.2022.113106

[27] Lim YH Jørgensen JT So R. Long-term exposure to road traffic noise and incident myocardial infarction: a Danish Nurse Cohort study. Environ Epidemiol (Philadelphia, Pa.). 2021;5:e148. 10.1097/EE9.0000000000000148 PMC8078417 33912785

[28] Bai L Shin S Oiamo TH. Exposure to road traffic noise and incidence of acute myocardial infarction and congestive heart failure: a population-based cohort study in Toronto, Canada. Environ Health Perspect. 2020;128:87001. 32783534 10.1289/EHP5809 PMC7422718

[29] Vienneau D Saucy A Schäffer B; SNC study group. Transportation noise exposure and cardiovascular mortality: 15-years of follow-up in a nationwide prospective cohort in Switzerland. Environ Int. 2022;158:106974. 34775186 10.1016/j.envint.2021.106974

[30] Pyko A Andersson N Eriksson C. Long-term transportation noise exposure and incidence of ischaemic heart disease and stroke: a cohort study. Occup Environ Med. 2019;76:201–207. 30804165 10.1136/oemed-2018-105333

[31] Eminson K Cai YS Chen Y. Does air pollution confound associations between environmental noise and cardiovascular outcomes? - A systematic review. Environ Res. 2023;232:116075. 37182833 10.1016/j.envres.2023.116075

[32] Björk J Strömberg U Rosengren A. Predicting participation in the population-based Swedish cardiopulmonary bio-image study (SCAPIS) using register data. Scand J Public Health. 2017;45(17_suppl):45–49. 28683666 10.1177/1403494817702326

[33] Segersson D Eneroth K Gidhagen L. Health impact of PM10, PM2.5 and black carbon exposure due to different source sectors in Stockholm, Gothenburg and Umea, Sweden. Int J Environ Res Public Health. 2017;14:742. 28686215 10.3390/ijerph14070742 PMC5551180

