# Supplementary material for: Road-traffic noise exposure and coronary atherosclerosis in the Swedish CArdioPulmonary bioImage Study (SCAPIS)
Source: Environ Epidemiol. 2024 Oct 3;8(5):e344. doi: 10.1097/EE9.0000000000000344 (PMC11452091; doi:10.1097/EE9.0000000000000344)

*Figure S1 Flowchart of study participants* ........................................................................................ 1
*Figure S2. Road traffic noise exposure per site among study subjects included in the main model.* ........... 2
*Table S1. Correlation matrix for exposure variables. All variables are calculated as a mean exposure 10 y before the enrollment.* ........................................................................................................ 2
*Figure S3. Odds ratios and 95% CI for atherosclerosis in relation to road-traffic noise per IQR increase during 10 y prior to the enrollment in sensitivity analyses involving different exposure times and additional adjustments* ........................................................................................................ 3

## Figure S1 Flowchart of study participants

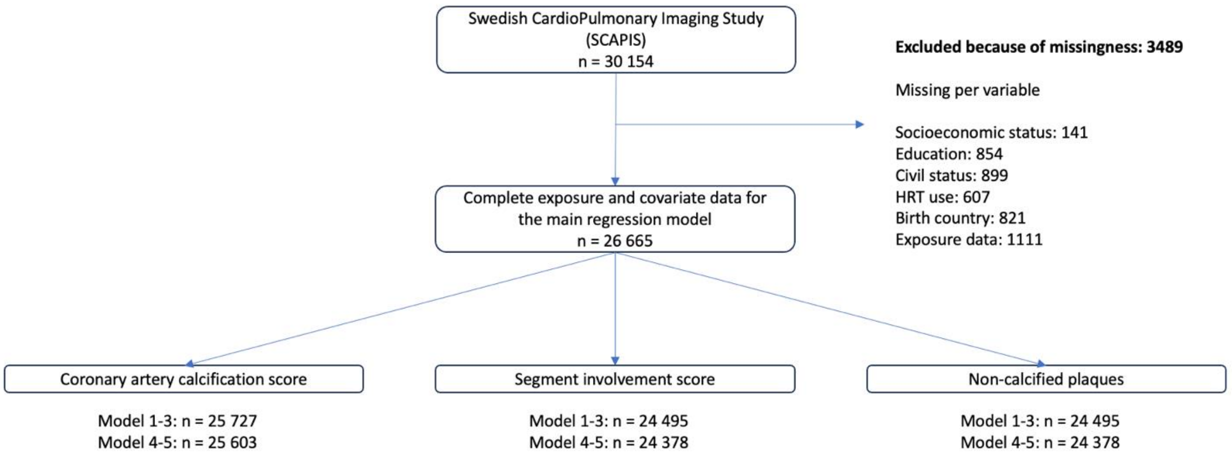

Figure S2. Road traffic noise exposure per site among study subjects included in the main model.

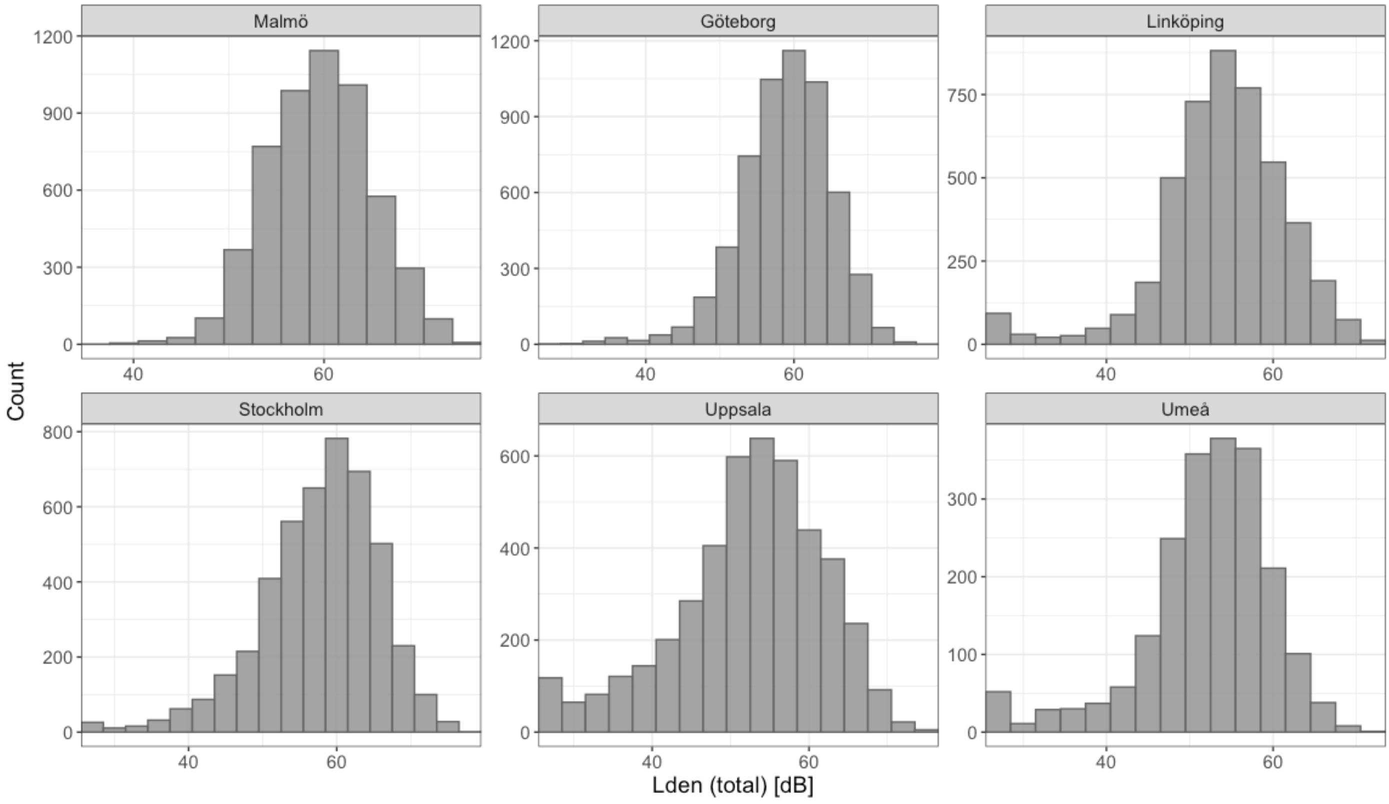


Table S1. Correlation matrix for exposure variables. All variables are calculated as a mean exposure 10 y before the enrollment.

| | Lden | $NO_2$ | $NO_x$ | $PM_{2.5}$ | NDVI |
|---|---|---|---|---|---|
| Lden | 1.00 | | | | |
| $NO_2$ | 0.66*** | 1.00 | | | |
| $NO_x$ | 0.67*** | 0.99*** | 1.00 | | |
| $PM_{2.5}$ | 0.43*** | 0.83*** | 0.79*** | 1.00 | |
| NDVI | -0.62*** | -0.70*** | -0.72*** | -0.51*** | 1.00 |

*** p < .001, ** p < 0.05, * p < 0.1

## Figure S3. Odds ratios and 95% CI for atherosclerosis in relation to road-traffic noise per IQR increase during 10 y prior to the enrollment in sensitivity analyses involving different exposure times and additional adjustments

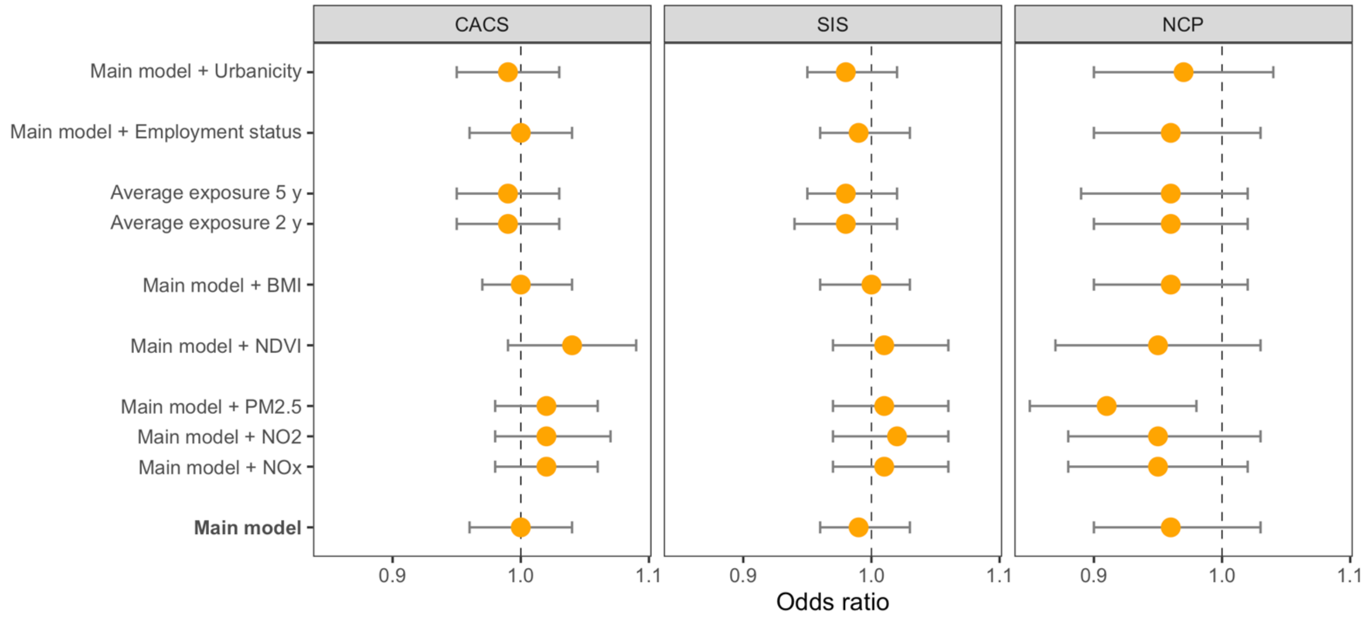

Supplement: Supplementary file 1 [file ee9-8-e344-s001.pdf]
